# Progress and challenges in integrated traditional Chinese and western medicine in China from 2002 to 2021

**DOI:** 10.3389/fphar.2024.1425940

**Published:** 2024-09-06

**Authors:** Man-Ci Zhou, Yu-Tong Fei, Xiao-Zhen Lai, Jian Lan, Bo Liu, Zhi-Wei Wang, Hai Fang, Jian-Ping Liu, Hong-Guo Rong

**Affiliations:** ^1^ Institute for Excellence in Evidence-Based Chinese Medicine, Beijing University of Chinese Medicine, Beijing, China; ^2^ School of Management, Beijing University of Chinese Medicine, Beijing, China; ^3^ Center for Evidence-Based Chinese Medicine, Beijing University of Chinese Medicine, Beijing, China; ^4^ School of Traditional Chinese Medicine, University of Chinese Medicine, Beijing, China; ^5^ China Center for Health Development Studies, Peking University, Beijing, China; ^6^ National Institute of Chinese Medicine Development and Strategy, Beijing, China

**Keywords:** integrative medicine, traditional Chinese medicine, western medicine, healthcare, China

## Abstract

**Objective:**

The World Health Organization advocated for enhanced integration of traditional medicine and complementary medicine into national healthcare systems across all countries. This study aims to explore the progress and challenges in integrated traditional Chinese and western medicine (ITCWM) in China over 20 years using nationwide data.

**Methods:**

This cross-sectional study examined various facets of ITCWM in China in terms of policies, resources, services, and funding. National policy documents from 2009 onwards were retrieved from official websites of the Chinese government. Data on ITCWM resources, services and subsidies from 2002 to 2021 were extracted from the China Statistical Yearbooks of Chinese Medicine and China Health Statistical Yearbooks. Research fund projects with the ITCWM discipline were collected from the database of National Natural Science Foundation of China. A mixed method of both quantitative and qualitative approaches was employed to present a comprehensive overview of the collected data.

**Results:**

This study presented five key findings. First, despite the issuance of 17 national policies by the Chinese government since 2009 to promote the development of traditional Chinese medicine (TCM), only three of them were specifically tailored for ITCWM. Second, although the average annual growth rates of ITCWM institutions, beds, and practitioners reached 0.35%, 10.56%, and 10.88% from 2002 to 2021, with more equitable allocation of ITCWM resources, the overall proportion of ITCWM remained below 5% in 2021. Third, progress has been made in ITCWM practices, yet service efficiency requires further enhancement. In 2021, ITCWM hospitals accounted for 2% of outpatient and emergency visits and 1.57% of hospital admissions among all hospitals, and 9.82% of delivered services were preventive healthcare services. Fourth, ITCWM served a crucial role in primary healthcare services, but its service capabilities need improvement. From 2007 to 2021, the average growth rates of ITCWM clinics, outpatient departments, and practitioners in outpatient departments were 13.30%, 2.57%, and 12.14%, respectively, while the proportion within TCM hospitals dropped. Lastly, despite the Chinese government’s emphasis on financial investment and related project funding for ITCWM, it remained lower than that allocated to TCM and western medicine.

**Conclusion:**

ITCWM played a pivotal role in China’s healthcare system to advance individuals’ health and well-being across the lifespan. In the future, China will provide further support to enhance ITCWM health resources and improve service capability, and the strategic integration of ITCWM into the broader healthcare system will play a crucial role in achieving universal health coverage and the Sustainable Development Goals.

## Introduction

During the first Traditional Medicine Global Summit in August 2023, the World Health Organization (WHO) unveiled a new vision for traditional medicine, emphasizing evidence-based integration as a crucial component. This vision underscored the importance for countries to seamlessly incorporate traditional and complementary medicine into their national healthcare systems (World Health, 2019). In China, the integrated traditional Chinese and western medicine (ITCWM) has been widely promoted and studied, showcasing distinctive advantages and significant contributions to achieving universal health coverage (Burki, 2023; Xie et al., 2024).

Recognizing the pivotal role of integration in the national healthcare system, the Chinese government has implemented a series of policy measures since the early 1950s. The policy of equal importance to TCM and western medicine thus became part of long-term national health policies in China (WENG et al., 2018). In 1994, the government formulated the Basic Standards for Medical Institutions, delineating the standards for ITCWM hospitals, outpatient departments, and clinics (Health tMo, 1994). Notably, tertiary ITCWM hospitals require over 60% of practitioners to specialize in ITCWM, with department heads holding deputy director or higher positions, and at least 40% skilled in ITCWM or TCM. In April 2009, the State Council issued *Opinions on deepening healthcare system reform*, (Yip et al., 2019) emphasizing equal emphasis on TCM and western medicine as a guiding principle for deepening the healthcare system reform. Furthering the integration, *Opinions on supporting and promoting the development of traditional Chinese medicine* was released in May 2009, manifesting the establishment of a complete policy system for TCM (China SCotPsRo, 2009b; China SCotPsRo, 2003). The Chinese government has continually sought suitable approaches to integrate TCM into the national healthcare system, resulting in a significant increase in the number of ITCWM institutions and practitioners.

Noteworthy achievements in ITCWM research included the 2015 Nobel laureate Youyou Tu’s isolation of artemisinin from a Chinese herbal medicine-sweet wormwood, which saved millions of lives worldwide, (Owens, 2015) highlighting the potential advantages of ITCWM (Tu, 2016). Edward Kelley, Director of the WHO Integrated Health Services Department, has emphasized the substantial contribution that Traditional, Complementary and Integrative Medicine (TCIM) can make to universal health coverage through primary healthcare and the provision of essential health services (Zhang et al., 2019). Nevertheless, integrating traditional and complementary medicine into the national healthcare system poses challenges. According to the 2014–2023 WHO strategy, Member States, including China, faced obstacles such as a lack of integrated research data, financial support, education and training, and regulatory frameworks (von Schoen-Angerer et al., 2023). India is a prime example of a nation that is actively striving to integrate biomedical and TCIM practices (Krishna, 2024; Chaturvedi et al., 2023). Despite efforts to revive traditional local health practices and integrate them into the Ayush system, indigenous practices are frequently overlooked and at risk of being marginalized (von Schoen-Angerer et al., 2023). In both Canada and the United States, TCIM is not included in the medical school curricula. TCIM is under professional regulation in specific Canadian provinces and is linked to regulatory colleges (Ng et al., 2023).

The World Health Assembly has decided to extend the current WHO Traditional Medicine Strategy until 2025, and develop a new 10-year strategy (Organization, 2023). Despite decades of ITCWM exploration in China, the integration is primarily at the physician level, with trained professionals able to treat patients in both traditional and western medicine contexts. There remains a gap in research depth, as available data analysis does not fully match the widespread use, complexity, and diversity of ITCWM in China. Leveraging national statistics on TCM over the past two decades, our aim is to explore the current situation and challenges in ITCWM in China, strengthen the role ITCWM plays in achieving health and well-being and universal health coverage (UHC), and provide evidence-based support for the upcoming 10-year strategy on traditional medicine by WHO.

## Methods

### Data sources

This cross-sectional study examined data related to ITCWM policies, practices, financial investments, and research fund projects. Rigorous efforts were made to maintain consistency across diverse data sources, each of which employed distinct collection instruments and methodologies.

The policy document data were retrieved from the official websites of the Chinese government, using the keywords “integrative medicine,” “integrated traditional Chinese and western medicine,” and “traditional Chinese medicine.” Since 2009, China has initiated a series of new medical reform measures. Since then, the government has gradually shifted focus to ITCWM. Therefore, the time span was set from January 2009 to April 2024. In addition, only national-level policy documents were included to ensure the analysis captured representative and effective national policies related to China’s ITCWM. Policy documents lacking practical content, guidance value, or a close relation to ITCWM were excluded.

Data on ITCWM practices from 2002 to 2021 were extracted from China Statistical Yearbooks of Chinese Medicine and China Health Statistical Yearbooks (Commission, 2002-2021; NAoTC, 2002-2021). Data regarding ITCWM financial investments (average financial aid income for hospitals) from 2002 to 2021 were obtained from China Statistical Yearbooks of Chinese Medicine (NAoTC, 2002-2021). The China Statistical Yearbooks of Chinese Medicine, operated by the National Administration of Traditional Chinese Medicine, served as the primary data source. This comprehensive database officially compiles statistics on the development of TCM in 31 provinces of the Chinese mainland from 1987 to 2021, and includes statistics on various facets of TCM development, such as health resources of TCM, operations and services of TCM healthcare institutions, education of TCM, scientific research of TCM, and financial allocations for TCM. Data from Hong Kong, Macao, and Taiwan were excluded due to inconsistent statistical standards. Research fund projects within the ITCWM discipline were sourced from the National Natural Science Foundation of China database (http://fund.zsci.com.cn/Index/index.html) for the period from 1 January 2002 to 31 December 2020 (NNSFo, 2002-2021).

### Statistical analysis

In this study, policy documents were analyzed using qualitative research methods. The trend of ITCWM-related policy issuance was analyzed using Excel 2019 (Microsoft, Redmond, WA, United States). Four core variables were considered: title, release date, issuing authority, and key points. Descriptive statistical methods were employed to analyze ITCWM practices, financial investments, and research fund projects. Categorical variables were presented using numbers and percentages. ITCWM practices encompassed institutions, bed capacity, practitioners (licensed TCM doctors, licensed assistant TCM doctors, and TCM pharmacists), outpatient services (outpatient emergency visits, health examinations, and family health services), inpatient services (admitted and discharged patients, turnover rate of hospital beds, occupancy rate of hospital beds, and average length of stay in hospital), TCM specialty services (patients receiving preventive TCM healthcare services, application of TCM medical technology, spatial size of TCM preparation rooms, diversity of TCM preparations, and presence of TCM diagnostic and therapeutic equipment valued at over 5,000 yuan). ITCWM funding was adopted to reflect the financial investment of ITCWM institutions and research fund projects within the ITCWM discipline.

Dagum Gini coefficient and kernel estimation were used to assess the equality of health resource allocation in ITCWM. Compared to conventional approaches like the Gini coefficient and the Theil index, it demonstrates superior performance and accuracy in addressing sub-regional imbalances. The Dagum Gini coefficient method is an effective way to measure the inequality in the development of ITCWM hospitals, health staff, and beds across different regions. The overall difference can be broken down into three components: intra-regional difference, interregional variation, and super-variable density contribution (Dagum, 1997). The study also used kernel density estimation to analyze the evolving patterns and absolute differences in the distribution of health resources in ITCWM institutions over time. This method helps to remove the uncertainty caused by unknown parameters and enables a more detailed exploration and discussion of spatial variances (Silverman, 1986; Scott, 2008). To reflect the availability of TCM healthcare resources, *per capita* metrics for ITCWM hospitals, beds and practitioners were calculated by dividing the total count for each indicator by the number of local population for the corresponding year. All data analyses were conducted using Stata version 13.0 (StataCorp).

## Results

### Policies on TCM development in China (2009–2023)

Supplementary Table S1 delineates the trajectory of 17 national policies on the development of TCM in China spanning from 2009 to 2023. The main body of policy issuance is characterized by the State Council as the core, with the participation of the National Administration of Traditional Chinese Medicine, the National Health Commission, and other departments. The key points of the policy have gradually changed from placing equal emphasis on TCM and western medicine to innovating collaborative medical models of ITCWM, strengthening the building of human resources, and establishing a quality management system for ITCWM.

In a significant move in March 2009, the State Council approved *Opinions on deepening healthcare system* reform, underscoring the necessity to place equal emphasis on both TCM and western medicine, and advocate for technological innovation in ITCWM (China SCotPsRo, 2009a). Subsequent to these foundational policies, China successively introduced seminal documents, including *Outline of the strategic plan on the development of traditional Chinese medicine (2016–2030)*, *Law of the People’s Republic of China on traditional Chinese medicine*, and *the 14th Five-Year Plan for the development of traditional Chinese medicine*. An important milestone in 2023 was the issuance of *Management measures for pilot projects on the construction of “flagship” hospitals for integrated traditional Chinese and western medicine.* This move heightened scrutiny over pilot projects to standardize and institutionalize management, thereby augmenting project implementation efficiency and effectiveness.

Presently, China has established a relatively comprehensive policy framework for TCM, providing a favorable policy environment and legal safeguards for TCM development. However, it is noteworthy that the majority of these policies are embedded within broader national frameworks, with only three policies specifically tailored for ITCWM. There is a lack of supportive policies for incentivizing and assessing ITCWM, the scope of practice of TCM and western medicine practitioners, and the training of ITCWM personnel.

### ITCWM resources

#### Trends in ITCWM healthcare institutions (2002–2021)

**FIGURE 1:**
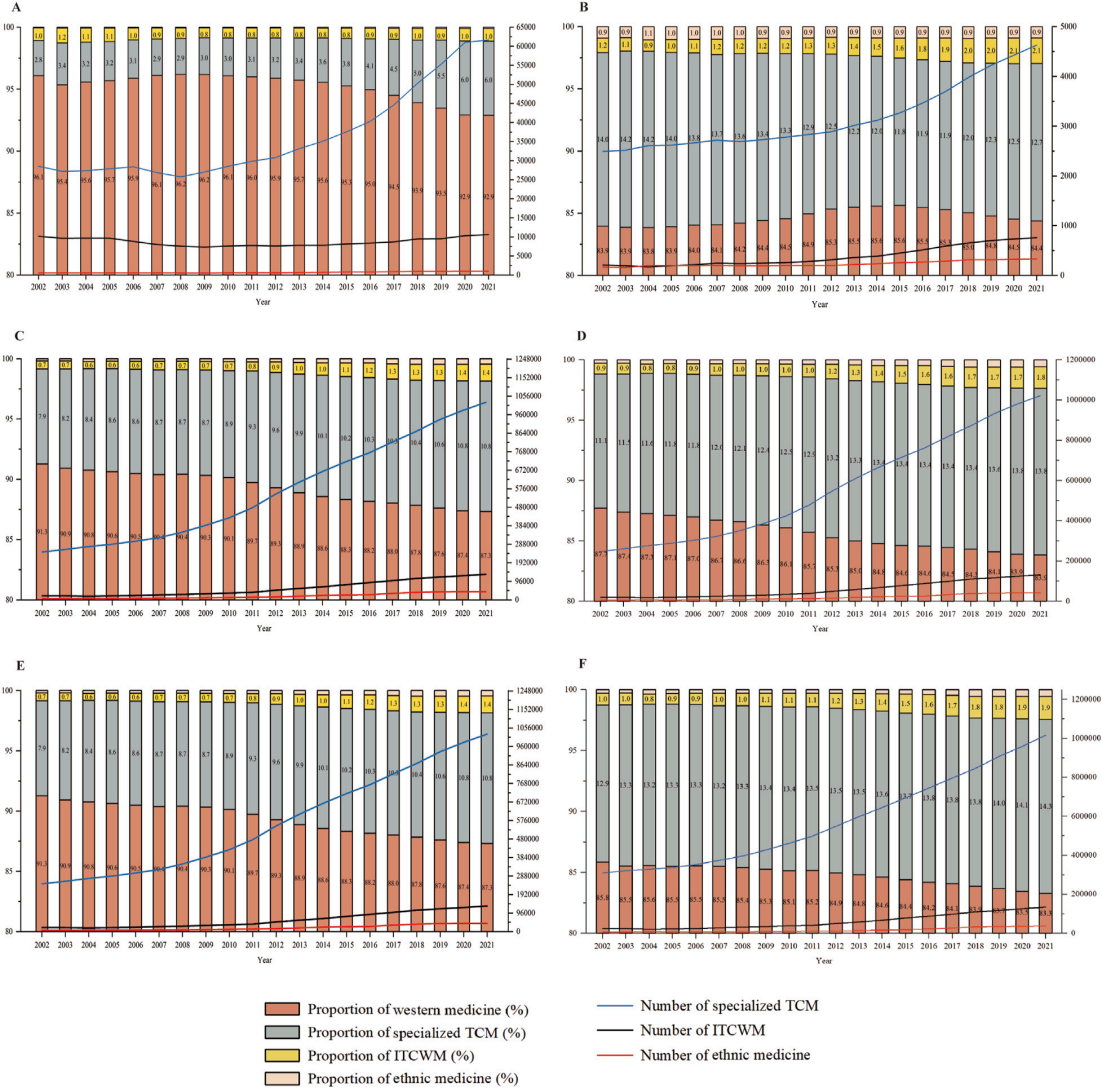
Number and share of health resources in different types of healthcare institutions, 2002–21 **(A)** Different types of healthcare institutions. **(B)** Different types of hospitals. **(C)** Beds in different types of healthcare institutions. **(D)** Beds in different types of hospitals. **(E)** Practitioners in different types of healthcare institutions. **(F)** Practitioners in different types of hospitals.

Figure 1 presents the year-by-year trends in the health resources of healthcare institutions. Healthcare institutions in China included both TCM and western medicine entities. Specifically, TCM healthcare institutions refer to specialized TCM, ITCWM, and ethnic medicine hospitals, as well as outpatient departments specializing in TCM, ITCWM, and ethnic medicine.

The presence of ITCWM healthcare institutions and ITCWM hospitals remained relatively low, suggesting that their overall scale and number have not yet reached a level comparable to that of TCM or western medicine. As shown in Figure 1A, as of 2021, the number of ITCWM healthcare institutions reached 10,617, comprising approximately 1.03% of the total count. The number of ITCWM hospitals, instead, exhibited consistent growth with an average growth rate of 2.99%, reaching 2.07% nationwide in 2021 (Figure 1B).

#### Trends in bed capacity in ITCWM healthcare institutions (2002–2021)

The bed count in ITCWM healthcare institutions experienced an increase from 20,583 (0.66%) in 2002 to 1,32,647 (1.40%) in 2021 (Figure 1C). Similarly, the number of hospital beds for ITCWM has displayed a consistent upward trajectory. With an annual growth rate of 12.47% from 2004 to 2021, ITCWM hospital beds constituted 1.78% (1,32,094) of the total hospital bed capacity in 2021 (Figure 1D).

#### Trends in practitioners in ITCWM healthcare institutions (2002–2021)

Figure 1E reports that the number of practitioners in ITCWM healthcare institutions has exhibited steady growth since 2012, with a count of 1,40,473 (1.28% of the total) in 2021. As for ITCWM hospitals, the number of practitioners in ITCWM hospitals reached 1,34,217 in 2021, and the share of practitioners increased from 0.97% in 2002 to 1.89% in 2021 (Figure 1F).

TCM practitioners primarily consist of licensed TCM doctors, licensed assistant TCM doctors, and TCM pharmacists. Table 1 illustrates the number and proportion of TCM practitioners in ITCWM hospitals. From 2002 to 2021, there was a notable increase in the count of licensed TCM doctors (from 1,845 to 17,227), licensed assistant TCM doctors (from 168 to 990), and TCM pharmacists (from 217 to 2,851). In 2021, the overall percentage of licensed TCM doctors, licensed assistant TCM doctors, and TCM pharmacists in ITCWM hospitals stood at 2.77%, 0.90%, and 2.06%, respectively. The data indicated that the number of TCM professionals in ITCWM hospitals has continued to grow, but there is still a need to enhance staffing further.

**TABLE 1:** Number and share of TCM practitioners in ITCWM hospitals, 2002–21.

Year	Licensed TCM doctors		Licensed assistant TCM doctors		TCM pharmacists	
	Total	ITCWM (%)	Total	ITCWM (%)	Total	ITCWM (%)
2002	1,80,227	1,845 (1.02)	33,692	168 (0.50)	18,304	217 (1.19)
2003	1,74,387	1,954 (1.12)	32,571	178 (0.55)	19,088	198 (1.04)
2004	1,71,419	1,931 (1.13)	30,934	161 (0.52)	19,643	203 (1.03)
2005	1,65,906	2,016 (1.22)	28,792	152 (0.53)	19,533	207 (1.06)
2006	1,66,614	1,965 (1.18)	28,514	138 (0.48)	21,324	270 (1.27)
2007	2,06,842	2,408 (1.16)	35,091	154 (0.44)	82,494	776 (0.94)
2008	2,18,044	2,721 (1.25)	35,189	195 (0.55)	88,673	840 (0.95)
2009	2,36,277	3,447 (1.46)	36,302	211 (0.58)	93,178	1,083 (1.16)
2010	2,56,361	4,077 (1.59)	37,743	251 (0.67)	97,100	1,120 (1.15)
2011	2,67,225	4,342 (1.62)	42,047	263 (0.63)	1,00,116	1,102 (1.10)
2012	3,05,372	5,461 (1.79)	51,407	353 (0.69)	1,07,630	1,384 (1.29)
2013	3,28,998	6,186 (1.88)	52,684	411 (0.78)	1,10,243	1,590 (1.44)
2014	3,54,973	7,103 (2.00)	63,600	426 (0.67)	1,11,991	1,678 (1.50)
2015	3,83,145	8,547 (2.23)	69,045	509 (0.74)	1,13,820	1,868 (1.64)
2016	4,09,275	9,983 (2.44)	72,315	521 (0.72)	1,16,622	2,028 (1.74)
2017	4,48,716	11,668 (2.60)	78,321	651 (0.83)	1,20,302	2,176 (1.81)
2018	4,89,582	12,798 (2.61)	85,872	759 (0.88)	1,23,913	2,350 (1.90)
2019	5,33,620	14,076 (2.64)	91,163	904 (0.99)	1,27,154	2,440 (1.92)
2020	5,78,091	15,529 (2.69)	1,04,679	988 (0.94)	1,31,163	2,676 (2.04)
2021	6,21,176	17,227 (2.77)	1,10,501	990 (0.90)	1,36,719	2,851 (2.06)

#### Equity of health resource allocation in ITCWM hospitals (2002–2021)

**FIGURE 2:**
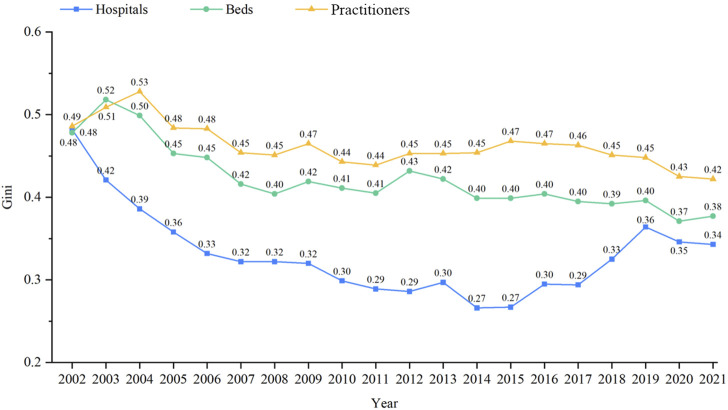
Gini coefficient of ITCWM health resources by population trend from 2002–21.

Figure 2 shows the Gini coefficient trend of ITCWM health resources per 10,000 population from 2002 to 2021. The Gini index of ITCWM hospitals showed fluctuating decline and rise, reaching its lowest point at 0.266 in 2014. The Gini coefficient consistently remained within the 0.2–0.4 range, indicating relatively equitable distribution. In contrast, when considering beds and practitioners, their Gini index decreased from 0.48 and 0.49 to 0.38 and 0.42, demonstrating an increase in fairness despite persisting disparities at relatively high levels. The decomposition of intra- and inter-regional differences in ITCWM health resources revealed large inter-regional disparities among eastern, central, and western provinces in terms of bed and practitioner resources (Gb 36.91% and 49.48% in 2021) (Supplementary Table S2).

**FIGURE 3:**
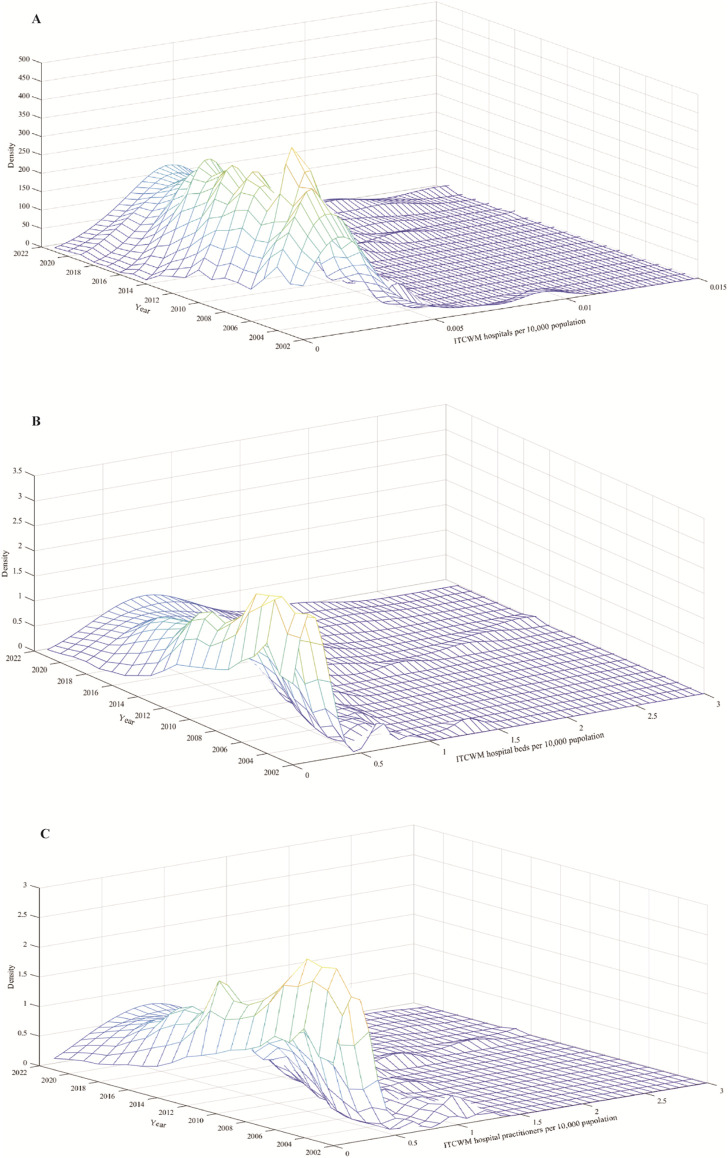
Kernel density estimation curve for ITCWM health resources **(A)** ITCWM hospital per 10,000 population. **(B)** ITCWM hospital beds per 10,000 population. **(C)** ITCWM hospital practitioners per 10,000 population.

Figure 3 illustrates the kernel density estimation curve for ITCWM hospitals, beds, and practitioners. From the distribution position, the center point of the Kernel density estimation map shifted to the right, signifying a rise in ITCWM healthcare resources available per 10,000 population. The right tails gradually were gradually lengthening, from 2002 to 2021, resulting in a wider interval span. This divergence implies an increasing gap between the highest and lowest levels of health resources available at ITCWM hospitals per 10,000 population.

### ITCWM services

#### Analysis of outpatient services in ITCWM hospitals (2007–2021)

**FIGURE 4:**
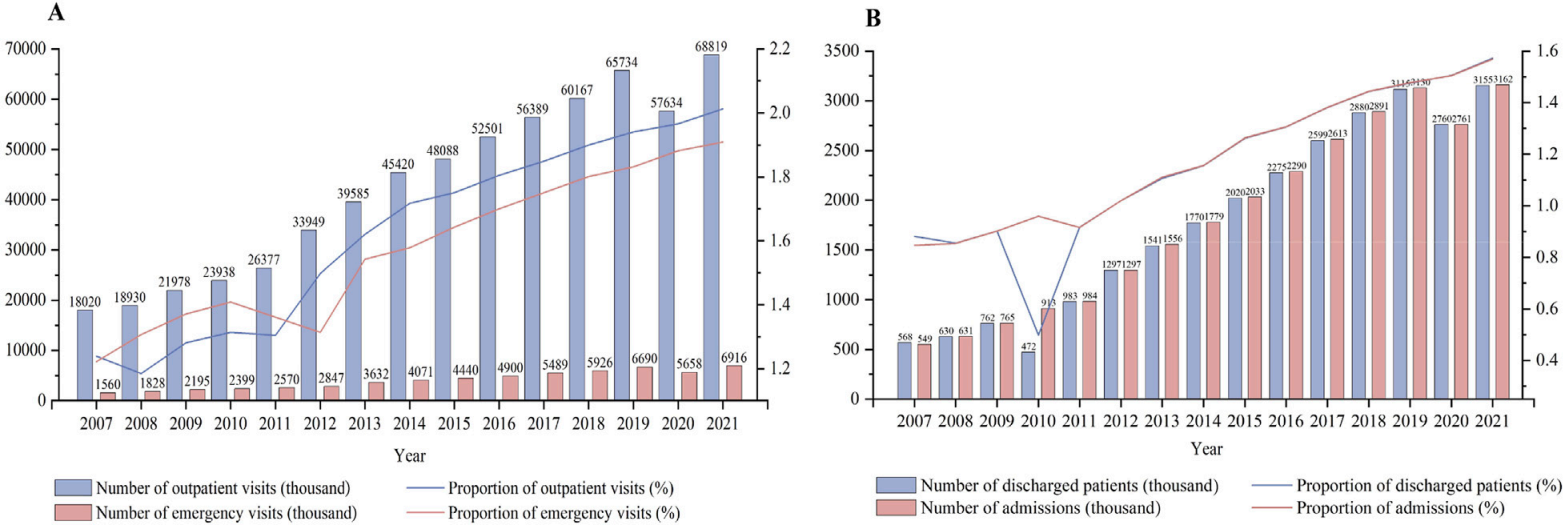
Changes of outpatient and inpatient services in ITCWM hospitals, 2007–21 **(A)** Outpatient and emergency visit. **(B)** Discharged patients and admissions.

Figure 4 shows the trends in outpatient and emergency visits, and inpatient visits in ITCWM hospitals from 2007 to 2021. Notably, there was a substantial increase in the number of outpatient and emergency visits, rising from 19,580,127 (1.24%) in 2007 to 75,735,249 (2.00%) in 2021, suggesting an improvement in service capability (Figure 4A). In Supplementary Table S3, the number of health examinations in ITCWM hospitals increased from 3,32,808 (0.89%) in 2003 to 7,488,913 (2.62%) in 2021.

Supplementary Table S4 shows the changes in outpatient prescriptions in ITCWM hospitals from 2012 to 2021. The number of TCM prescriptions showed a continual increase, reaching 29,057,131 by 2021, constituting 43.27% of the overall prescriptions. The proportion of antimicrobial prescriptions in ITCWM hospitals has consistently remained below the national average, indicating standardized and rational use of antimicrobial agents in these hospitals despite an upward trend.

#### Analysis of inpatient services in ITCWM hospitals (2007–2021)

As shown in Figure 4B, the number of admissions exhibited a consistent upward trajectory until 2019, reaching 3,161,964 (1.57% of the total) in 2021. Concurrently, the number of discharged patients experienced an initial dip in 2010 but steadily increased until 2019, with the 2021 proportion representing 1.57% of the total discharges. It seems that despite the recent growth in inpatient and outpatient services at ITCWM hospitals in China, the level of medical technology and public trust is not as high as that in general hospitals.

Supplementary Table S5 details the progressive rise in the turnover rate of ITCWM hospital beds, escalating from 19.53% in 2003 to 28.20% in 2021. Notably, this rate was 3.98 times lower than that observed in general hospitals. The share of ITCWM hospital bed occupancy rate exhibited an increasing trend followed by a subsequent decrease, culminating at 71.02% in 2021. The average length of hospital stay in ITCWM hospitals was 9.9 days in 2021, marking a reduction of 1.41 days compared to 2003, indicating an enhancement in the efficiency and quality of healthcare services.

#### Analysis of TCM specialty services in ITCWM hospitals (2012–2021)

Table 2 delineates the changes in TCM specialty services in ITCWM hospitals from 2012 to 2021. In 2021, there was a noticeable increase in the number of patients receiving preventive TCM healthcare services, application of TCM medical technology, spatial size of TCM preparation rooms, diversity of TCM preparations, and presence of TCM diagnostic and therapeutic equipment valued at over 5,000 yuan in ITCWM hospitals, compared to 2020. However, TCM hospitals still primarily deliver distinctive TCM services.

**TABLE 2:** Changes in TCM specialty services in ITCWM hospitals, 2012–21.

Year	Number of patients receiving preventive TCM treatment services		Average number of TCM medical technology applied		Spatial size of TCM preparation rooms		Average number of diversified TCM preparations per hospital		Number of TCM diagnostic and therapeutic equipment over 5,000 yuan	
	TCM hospitals	ITCWM hospitals (%)	TCM hospitals	ITCWM hospitals	TCM hospitals (m^2^)	ITCWM hospitals (m^2^)	TCM hospitals	ITCWM hospitals	TCM hospitals	ITCWM hospitals
2012	56,974,357	2,321,350 (4.07)	N/A	N/A	6,24,656	29,810	N/A	N/A	91,021	4,575
2013	19,701,219	1,021,020 (5.18)	N/A	N/A	7,44,303	39,793	N/A	N/A	1,13,594	6,119
2014	16,209,256	1,061,992 (6.55)	N/A	N/A	3,425,067	62,873	N/A	N/A	55,459	8,066
2015	16,056,643	1,077,241 (6.71)	37	28	7,84,320	40,369	59	113	1,33,328	8,722
2016	17,051,312	1,181,711 (6.93)	19	10	8,87,252	80,085	66	90	1,48,754	10,516
2017	19,829,791	1,492,040 (7.52)	36	29	8,95,732	58,763	70	68	1,63,417	13,009
2018	20,155,791	1,830,290 (9.08)	35	27	9,49,234	49,732	85	124	1,94,904	15,599
2019	20,115,387	1,701,107 (8.46)	36	26	9,79,646	56,249	79	112	2,11,497	17,378
2020	20,873,136	1,587,823 (7.61)	34	25	1,092,721	73,057	83	109	2,43,393	21,549
2021	23,371,493	2,294,377 (9.82)	34	26	1,223,998	83,161	90	138	2,65,454	24,581

N/A, not available.

#### Analysis of TCM resources in western healthcare institutions in 2021

Table 3 presents the integration status of TCM practices in western healthcare institutions in 2021. Notably, 44,118 (11.08%) healthcare institutions incorporated TCM clinical departments, primarily observed in township health centers, community health service centers, and general hospitals. Accompanying this, there were 3,08,392 (3.67%) beds dedicated to TCM services, supported by 4,18,651 (11.19%) licensed (assistant) TCM doctors and 79,123 (18.9%) TCM pharmacists. In 2021, TCM clinical departments in western healthcare institutions facilitated 301,988,000 (4.85%).

**TABLE 3:** Number and share of TCM health practices in western healthcare institutions, 2021.

Types	Institutions with clinical departments in TCM, N (%)	Beds in clinical departments of TCM, N (%)	Licensed (assistant) TCM doctors, N (%)	TCM pharmacists, N (%)	Outpatient and emergency visits, N in ten thousand (%)	Discharged patients, N in ten thousand (%)
Total	44,118 (11.08)	308,392 (3.67)	4,18,651 (11.19)	79,123 (18.9)	30198.8 (4.85)	648.37 (2.97)
General hospital	4,110 (21.41)	1,42,415 (3.03)	1,31,148 (7.93)	32,374 (15.64)	10499.32 (3.77)	316.62 (2.14)
Specialized hospital	401 (4.37)	27,311 (1.95)	26,989 (8.82)	6,102 (14.9)	862.55 (2.1)	42.98 (2.05)
Community health service center	5,026 (66.9)	17,785 (7.44)	40,631 (21.11)	9,192 (25.17)	6878.6 (9.88)	24.85 (7.81)
Community health station	4,543 (36.69)	971 (7.72)	15,790 (29.86)	1,812 (33.16)	1407.52 (10.05)	0.32 (5.66)
Township health center	18,609 (55.12)	1,11,512 (7.87)	95,506 (18.18)	18,651 (23.03)	9731.32 (8.38)	255.7 (7.94)
Specialized disease prevention and treatment center	38 (4.74%)	204 (0.50)	945 (6.88%)	341 (14.26)	32.96 (1.73)	0.23 (0.65)
Maternity and childcare center	933 (33.84)	2,600 (1.00)	10,264 (6.44)	2,480 (13.39)	629.46 (2.05)	4.13 (0.45)
Village clinic	N/A	N/A	44,048 (18.55)	812 (20.72)	N/A	N/A
Others	10,458	5,594	53,330	7,359	157.06	3.53

N/A, not available.

#### ITCWM practices in primary healthcare facilities (2002–2021)

**FIGURE 5:**
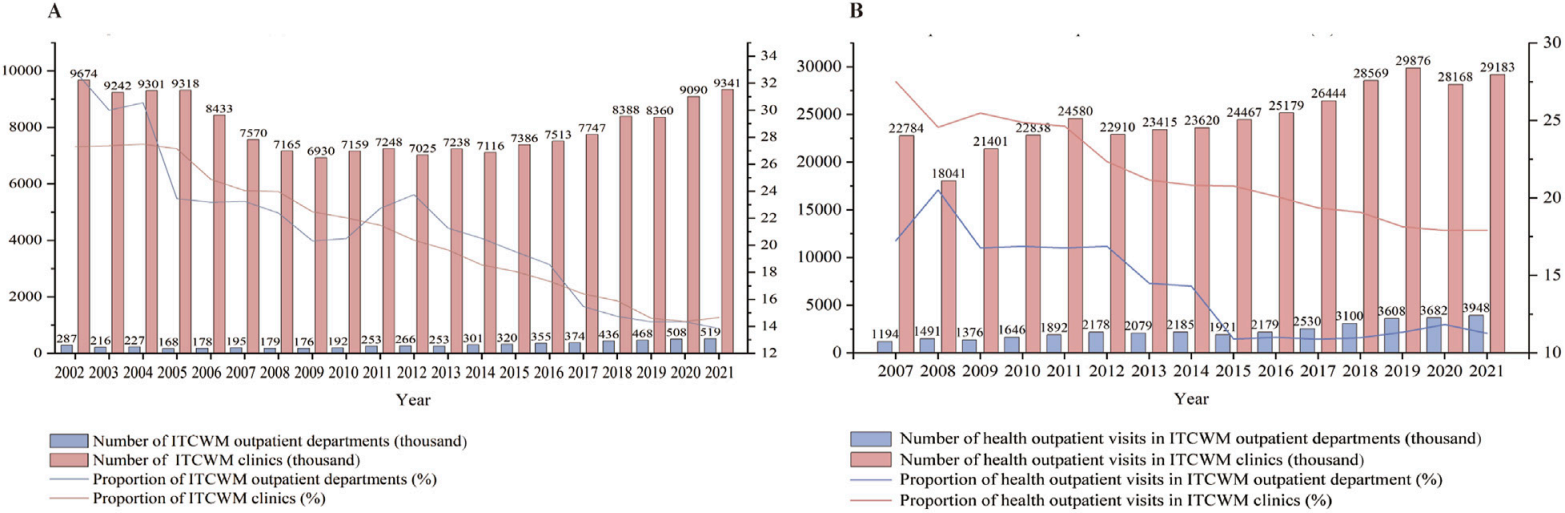
Changes of ITCWM practices in ITCWM primary healthcare facilities, 2002–21 **(A)** Level and share of ITCWM primary healthcare facilities. **(B)** Level and share of outpatient visits in ITCWM primary healthcare facilities.

Figure 5A shows the shifts in ITCWM primary healthcare facilities from 2002 to 2021. Since 2002, although the number of ITCWM outpatient departments increased from 287 to 519, the corresponding share experienced a decline, dropping from 32.88% to 13.83%. In 2021, ITCWM clinics represented 14.65% of the total primary healthcare facilities landscape. As for practitioners, ITCWM outpatient departments accommodated a total of 6,256 (15.05%) health practitioners in 2021 (Supplementary Table S6). From 2002 to 2021, there was a decrease of 4.72% in the proportion of licensed doctors and a 9.66% decline in licensed assistant doctors within ITCWM outpatient departments.

Figure 5B illustrates the trends in outpatient visits at ITCWM primary healthcare facilities from 2007 to 2021. The outpatient visits at ITCWM outpatient departments witnessed a notable rise, escalating from 1,194 thousand in 2007 to 3,948 thousand in 2021, constituting 11.27% of the total TCM outpatient departments. Furthermore, in 2021, outpatient visits in ITCWM clinics represented 17.91% of the total TCM clinics.

### ITCWM funding

#### Government subsidies for ITCWM hospitals

Supplementary Figure S1 depicts the government subsidies in TCM hospitals from 2004 to 2021. During this period, government subsidies per ITCWM hospital increased from 163.89 thousand USD in 2004 to 1435.88 thousand USD in 2021. ITCWM hospitals received lower subsidies compared to TCM and ethnic medicine hospitals. This indicated that there is significant room for increased investment in the health services.

#### Research fund projects for ITCWM discipline supported by NSFC

Supplementary Table S7 illustrates the changes in ITCWM discipline projects supported by NSFC from 2002 to 2020. The number of projects and their respective funding have seen a substantial increase, rising from 57 (1.65%) and 1563.24 thousand USD (0.81%) in 2002 to 342 (9.89%) and 20092.23 thousand USD (10.44%) in 2021. On an annual basis, the average funding for ITCWM discipline projects stands at 55619.03 thousand USD, lower than the average funding level for NSFC projects, which is 71181.76 thousand USD.

## Discussion

To our knowledge, this study represents the first comprehensive assessment of the development process in ITCWM in China over a span of two decades, utilizing national statistical data. Aligned with the principle of equal emphasis on TCM and western medicine, the Chinese government has strategically employed various measures to integrate ITCWM into the national healthcare system. These measures include facilitating equitable access to safe, high-quality, and effective ITCWM resources and services, manifesting the distinctive advantages of TCM, and advancing TCM disease prevention initiatives.

With steadfast support from national policies, the integration in China has undergone extensive exploration, yielding remarkable accomplishments. First, the total quantity of ITCWM health resources has experienced sustained growth, suggesting that ITCWM is gaining wider acceptance and integration into China’s healthcare system. Over the past two decades, there has been a substantial increase in ITCWM resources, including healthcare institutions, beds, and practitioners. Second, significant strides have been made in reducing regional disparities in the allocation of ITCWM resources across China, indicating a commitment to fostering greater equity, accessibility, and convenience in ITCWM health services. Decreased Gini coefficients indicated a reduction in regional disparity, aligning with findings from previous studies (Xu et al., 2022; Dai et al., 2022). Third, ITCWM has emerged as a crucial force driving the healthcare system reforms in China given the persistent expansion of ITCWM service capabilities and the distinctive advantages of TCM. The number of outpatient and emergency visits at ITCWM hospitals surged from 19,580,127 in 2007 to 75,735,249 in 2021. Concurrently, family health services expanded, and the length of hospital stays witnessed a discernible diminution, leading to decreased medical expenses, optimized resource utilization, and enhancement of overall health status (Xiang et al., 2023; Schneuer et al., 2023).

The unique advantages of TCM in disease prevention and health services are also evident. To enhance the core competitiveness, it became imperative to preserve and leverage the distinctive advantages of TCM (Dai et al., 2022). China has proactively promoted the concept of TCM disease prevention, implementing a health promotion project featuring preventative treatment of diseases (China SCotPsRo, 2022; China SCotPsRo, 2023a). In 2021, ITCWM hospitals provided preventive treatment of disease and health examinations for 2,294,377 and 7,488,913 times, respectively, reflecting a 44.50% and 55.17% surge from the preceding year. Lastly, ITCWM serves a crucial role in China’s primary healthcare service system. The findings highlight a consistent increase in the number of ITCWM clinics, outpatient departments, and practitioners. Notably, outpatient visits to ITCWM outpatient departments increased to 3.95 million in 2021. This has significantly contributed to higher equity, convenience, and accessibility of healthcare services for the general population (China SCotPsRo, 2023b).

The WHO has encouraged that all nations with TCIM integrated into their national healthcare systems. In recent years, China has continued to improve the system of ITCWM, bolster the development of ITCWM institutions, improve ITCWM services in general hospitals, and strive for a more equitable distribution of high-quality resources. The role of ITCWM in China’s healthcare system is becoming increasingly important. Japan, with a notably large elderly population, actively combines modern biomedicine and traditional medicine within its comprehensive national healthcare system (Yoshino et al., 2023). In China, earning the title of ITCWM doctor involves graduating from a university that focuses on TCM. However, in Japan, this qualification is not recognized, and one needs to graduate from a medical school that emphasizes conventional biomedicine (Ito et al., 2021).

Despite commendable achievements, challenges persist in China’s ITCWM landscape. First, national policies on ITCWM were often amalgamated with broader policies, resulting in a scarcity of dedicated national policies targeting ITCWM. As of 2023, only three national policies have been crafted exclusively for ITCWM, primarily focusing on the establishment of ITCWM medical centers and flagship hospitals (China SAoTCMotPsRo, 2011; China SAoTCMotPsRo, 2023; National Health Commission, 2021). Without clear national policies and support, practitioners may face challenges in the maldistribution of services and limited access to training programs (Al-Worafi and Al-Worafi, 2023). This also can lead to inconsistencies and lack of standardization in ITCWM practices, affecting patient outcomes and satisfaction. Future efforts should prioritize the formulation of policies, laws, and regulations tailored to the unique features of ITCWM (von Schoen-Angerer et al., 2023; Wong et al., 2012).

Second, China grapples with an overall shortage of ITCWM health resources and enduring disparities in ITCWM development, underscoring the need for policymakers to increase funding for research, education, and infrastructure development to ensure equitable access to ITCWM services. Externally, despite substantial growth in ITCWM healthcare facilities over the past 20 years, they still constitute less than 5% of the total healthcare infrastructure. The preference of patients for TCM or Western medicine, as well as government support for ITCWM, are key factors in the development of ITCWM. Existing research has shown that patients’ choice between TCM and Western medicine is influenced by five factors: the distinct characteristics of TCM and Western medicine, policy-related elements, patient understanding, and the effects of educational, and promotional efforts (Zhao et al., 2023). Concurrently, there is a lack of understanding regarding how TCM influences disease and the recovery process, hindering the progress of combining western medicine and TCM (Fu et al., 2021). Therefore, future efforts should focus on enacting appropriate legislation and regulatory frameworks, investigating the pharmacological mechanisms of traditional Chinese medicinal preparations, and enhancing public recognition and acceptance of ITCWM. Internally, the distribution of beds and practitioners in ITCWM hospitals remains uneven in the eastern, western, and central regions of China. This imbalance is closely linked to regional economic development levels and urban-rural dual structure (Dai et al., 2022; Shi et al., 2020; Zhang et al., 2021). To rectify this, it is imperative to increase government involvement, elevate ITCWM’s significance in economic and social development, and provide additional financial support to amplify ITCWM health resources (China SCotPsRo, 2021). Meanwhile, it is essential to improve the effectiveness of ITCWM services, strengthen the cultivation of highly skilled ITCWM talents, and increase research investments in the ITCWM field.

Third, the community-level ITCWM healthcare system remains incomplete, with relatively weak service capabilities. Efforts should be made to increase funding for community-level ITCWM initiatives, establish explicit service delivery protocols, and facilitate inter-disciplinary training programs to cultivate a competent workforce. Over the past two decades, the proportion of ITCWM clinics and outpatient departments within TCM has dwindled from 27.3% and 32.9% to 14.65% and 13.83%, respectively. Simultaneously, in community health service centers and township health clinics, the proportion of TCM practitioners falls short of the recommended 25%, standing at 21.10% and 18.80% (China SAoTCMotPsRo, 2022). Possible reasons include a shortage of in community-level ITCWM talents and limited government resource allocation (Ma et al., 2020; Ding et al., 2021). To address these issues, it is suggested that opportunities be created for western medicine practitioners to learn from their TCM counterparts, encouraging the involvement of western-trained personnel in TCM services, and boosting the diagnostic and treatment capabilities of TCM in primary healthcare settings. Furthermore, it was found that most practitioners and beds have been allocated to ITCWM hospitals, despite of the much larger number of primary healthcare facilities. This can be attributed to policymakers’ emphasis on prioritizing the development of large-scale ITCWM institutions, thereby overlooking the crucial role of primary healthcare. Patients also showed preference for seeking medical attention at hospitals rather than primary healthcare facilities, posing a risk of enlarging the capacity gap between hospitals and primary care institutions (Zhang et al., 2017; Yang et al., 2023). While it was important for the government to intervene in the development of policies that ensure proper integration within primary healthcare by reallocating health resources and standardizing ITCWM services, it was also necessary to intervene regarding the kinds of services or job types that can be linked or integrated with the ITCWM services at primary healthcare facilities. This strategic move is essential for bridging the capacity gap and ensuring the effective strengthening of primary care services.

Several limitations should be acknowledged in this study. First, the selection of indicators was restricted by data availability, with certain metrics such as patient burden and number of ITCWM practitioners not included in our analysis. Second, the study focused primarily on practices, excluding ITCWM education, industry, and other related areas, which might limit the comprehensiveness when assessing the overall development of ITCWM in China. Finally, the use of household registration population data may result in an overestimation of *per capita* health resources in economically developed regions and an underestimation in economically underdeveloped regions due to the typical migration of populations from underdeveloped to developed areas.

In conclusion, this study comprehensively analyzed the 20-year development of ITCWM in China. The Chinese government has undertaken a set of measures to integrate ITCWM into the country’s healthcare system, aiming to enhance equitable access to ITCWM resources and services. Despite these efforts, challenges persist, including insufficient health resources and imbalanced development of ITCWM. In the future, China should offer additional support to enhance ITCWM practices, explore optimal strategies to promote integration and leverage its role in advancing universal health coverage and improving population well-being.

## Data Availability

The datasets presented in this study can be found in online repositories. The names of the repository/repositories and accession number(s) can be found in the article/Supplementary Material.
