# Supplementary material for: Progress and challenges in integrated traditional Chinese and western medicine in China from 2002 to 2021
Source: Front Pharmacol. 2024 Sep 6;15:1425940. doi: 10.3389/fphar.2024.1425940 (PMC11412861; doi:10.3389/fphar.2024.1425940)
Supplement: Supplementary file 1 [file Table1.DOCX]

**Supplementary Materials**

**Supplemental Table 1: Summary of key reform policies for TCM in China, 09-2023**

| No. | Released date | Policy | Issuing authority | Key points |
| --- | --- | --- | --- | --- |
| 1 | March 7, 2009 | Opinions on deepening healthcare system reform | State Council (*Zhongfa* [2009] No 6) | ·Place equal emphasis on TCM and western medicine  ·Encourage scientific and technological innovation in the field of ITCWM research |
| 2 | May 7, 2009 | Opinions on supporting and promoting the development of traditional Chinese medicine | State Council (*Guofa* [2009] No 22) | ·Place equal emphasis on TCM and western medicine  ·Encourage exchanges between TCM and western medicine  ·Strengthen the cultivation of doctors with proficient expertise in both TCM and western medicine |
| 3 | June 2,2011 | Guidelines for integrated traditional Chinese medicine and western medicine hospital | National Administration of Traditional Chinese Medicine (*Guofa* [2009] No 22) | ·Propose requirements for the management and construction of ITCWM hospitals |
| 4 | February 2, 2016 | Outline of the strategic plan on the development of traditional Chinese medicine (2016-2030) | State Council (*Guofa* [2016] No 15) | ·Place equal emphasis on TCM and western medicine  ·Diagnosis and treatment plan of ITCWM  ·Working mechanism of ITCWM on the treatment and control of major difficult and complicated diseases  ·Construction of ITCWM hospitals  ·Cultivation of doctors with proficient expertise in both TCM and western medicine |
| 5 | October 25, 2016 | Outline of the Healthy China 2030 Plan | State Council (*Guofa* [2016] No 32) | ·Place equal emphasis on TCM and western medicine  ·Set up integrated health service system  ·Strengthen research on TCM for the prevention and treatment of common diseases |
| 6 | December 25, 2016 | Law of the People's Republic of China on traditional Chinese medicine | State Council | ·Mutual learning between TCM and western medicine  ·ITCWM education  ·Cultivate high-level ITCWM talents  ·Strengthen ITCWM research |
| 7 | October 20, 2019 | Guiding opinions on promoting the technology innovation in healthcare service of traditional Chinese medicine | State Council (*Guofa* [2019] No 31) | ·Utilize the advantages of TCM and ITCWM in health preservation, prevention, and treatment of chronic diseases  ·Establish the consultation system of ITCWM  ·Encourage exchanges between TCM and western medicine |
| 8 | January 22, 2021 | Several policy measures for accelerating the characteristic development of traditional Chinese medicine | General Office of the State Council (*Guobanfa* [2021] No 3) | ·Joint research on the treatment and control of major difficult and complicated diseases and infectious diseases  ·Encourage exchanges between TCM and western medicine |
| 9 | June 10, 2021 | Opinions on further strengthening traditional Chinese medicine work in comprehensive hospitals and promoting the coordinated development of traditional Chinese and western medicine | National Health Commission (*Guoweiyihan* [2021] No 126) | ·Improve ITCWM system in general hospitals  ·Innovative collaborative medical model of Chinese and western medicine  ·Cultivate high-level ITCWM talents |
| 10 | November 2, 2021 | Standards for the establishment of national integrated traditional Chinese and western medicine medical Centers (general hospitals) | National Health Commission (*Guoweibanyihan* [2021] No 544) | ·Develope standards for the establishment of national ITCWM centers (general hospitals) |
| 11 | March 1, 2022 | Law of the people’s republic of China on medical practitioners |  | ·Clarify the scope of TCM physician and ITCWM physician  ·Improve the education system for mutual learning between TCM and western medicine  ·Cultivate high-level ITCWM talents  ·Improve the diagnosis and treatment plans and combined use of TCM and western medicine |
| 12 | March 3, 2022 | The 14th Five-Year Plan for the development of traditional Chinese medicine | State Council (*Guobanfa* [2022] No 5) | ·Promote the coordinated development of ITCWM in general hospitals  ·Strengthen the service capacity construction of ITCWM hospitals  ·Improve the level of TCM services in relevant healthcare institutions |
| 13 | July 18, 2022 | Guiding Opinions on Further Promoting the Development of combined medical and elderly care | National Health Commission (*Guoweilaolingfa* [2022] No 25) | ·Utilize the advantages of ITCWM in health preservation, prevention, and treatment of chronic diseases  ·Promote TCM into families, communities, and institutions |
| 14 | January 18, 2023 | Implementation plan for strengthening traditional Chinese medicine elderly health services | (*Guozhongyiyaozongjiehehan* [2022] No 351) | ·Carry out and develop joint diagnosis and treatment of ITCWM  ·Develop clinical practice guidelines of ITCWM. |
| 15 | February 10, 2023 | Implementation plan for the major Projects of revitalizing and developing traditional Chinese medicine | State Council (*Guobanfa* [2023] No 3) | ·Strengthen the coordinated development of ITCWM projects: innovative construction of ITCWM model, collaborative construction of ITCWM clinical practices for major difficult and complicated diseases |
| 16 | May 23, 2023 | Theme activity plan for improving medical experience and enhancing patient experience (2023-2025) | (*Guoweiyizhengfa* [2023] No 11) | ·Promote ITCWM medical model in outpatient clinics  ·Create ITCWM teams  ·Carry out joint diagnosis and treatment of TCM |
| 17 | May 24, 2023 | Management measures for pilot projects on the construction of 'flagship' hospitals for integrated traditional Chinese and Western medicine | (*Guoweiyiyaozongjiehegfa* [2023] No 4) | ·Specific requirements for constructing ITCWM flagship hospitals |

**Supplemental Table 2: Decomposition of differences in Gini coefficients of ITCWM health resources, 2002-21**

| **Year** | **Hospitals** | | | **Beds** | | | **Practitioners** | | |
| --- | --- | --- | --- | --- | --- | --- | --- | --- | --- |
|  | **Gw** | **Gb** | **Gt** | **Gw** | **Gb** | **Gt** | **Gw** | **Gb** | **Gt** |
| 2002 | 32.42% | 20.64% | 46.94% | 33.27% | 10.66% | 56.07% | 32.37% | 26.91% | 40.72% |
| 2003 | 31.88% | 19.70% | 48.42% | 34.41% | 11.23% | 54.37% | 33.62% | 20.86% | 45.52% |
| 2004 | 31.81% | 11.14% | 57.05% | 32.19% | 36.19% | 31.62% | 31.30% | 46.79% | 21.91% |
| 2005 | 32.94% | 6.67% | 60.39% | 32.41% | 30.62% | 36.97% | 31.70% | 42.88% | 25.42% |
| 2006 | 33.81% | 5.02% | 61.17% | 31.96% | 37.04% | 31.01% | 31.24% | 45.58% | 23.19% |
| 2007 | 32.09% | 20.24% | 47.67% | 32.42% | 30.34% | 37.24% | 31.07% | 43.26% | 25.67% |
| 2008 | 32.83% | 15.99% | 51.18% | 31.54% | 30.06% | 38.40% | 30.94% | 40.94% | 28.12% |
| 2009 | 33.02% | 11.98% | 55.00% | 31.33% | 34.45% | 34.22% | 31.48% | 40.47% | 28.05% |
| 2010 | 33.82% | 13.40% | 52.78% | 30.30% | 41.10% | 28.60% | 30.34% | 44.69% | 24.97% |
| 2011 | 33.69% | 17.18% | 49.14% | 30.38% | 36.09% | 33.54% | 29.90% | 44.85% | 25.26% |
| 2012 | 33.36% | 18.17% | 48.47% | 31.22% | 42.09% | 26.69% | 30.16% | 44.53% | 25.32% |
| 2013 | 33.59% | 23.58% | 42.83% | 30.04% | 51.18% | 18.78% | 28.37% | 53.59% | 18.05% |
| 2014 | 33.18% | 26.52% | 40.30% | 29.47% | 53.72% | 16.81% | 27.97% | 58.07% | 13.97% |
| 2015 | 32.97% | 21.31% | 45.72% | 30.20% | 49.07% | 20.73% | 28.37% | 57.00% | 14.63% |
| 2016 | 33.00% | 17.87% | 49.13% | 31.84% | 47.61% | 20.55% | 30.41% | 52.68% | 16.91% |
| 2017 | 33.42% | 18.23% | 48.35% | 32.26% | 46.28% | 21.46% | 30.92% | 51.79% | 17.29% |
| 2018 | 33.95% | 24.70% | 41.35% | 33.05% | 41.77% | 25.18% | 31.48% | 51.09% | 17.43% |
| 2019 | 34.67% | 17.56% | 47.77% | 32.98% | 40.04% | 26.99% | 31.64% | 49.95% | 18.41% |
| 2020 | 34.28% | 10.63% | 55.09% | 32.78% | 39.14% | 28.08% | 31.27% | 50.67% | 18.06% |
| 2021 | 33.75% | 11.91% | 54.34% | 33.26% | 36.91% | 29.83% | 31.85% | 49.48% | 18.67% |

Abbreviation: ITCWM, integrated traditional Chinese and western medicine; Gw, intra-regional difference contribution value; Gb, inter-regional difference contribution value; Gt, transvariation intensity contribution value.

**Supplemental Table 3: The number of outpatient visits in ITCWM hospitals, 2003-21**

| **Year** | **Number of health examinations** | | **Number of family health services** | |
| --- | --- | --- | --- | --- |
|  | **Total** | **ITCWM**  **(%)** | **Total** | **ITCWM**  **(%)** |
|  |  |  |  |  |
| 2003 | 37,468,146 | 332,808 (0.89) | N/A | N/A |
| 2004 | 44,727,124 | 537,453 (1.20) | N/A | N/A |
| 2005 | 56,115,346 | 596,305 (1.06) | N/A | N/A |
| 2006 | 61,061,631 | 679,508 (1.11) | N/A | N/A |
| 2007 | 92,576,326 | 1,162,929 (1.26) | 5,897,627 | 44,449 (0.75) |
| 2008 | 100,660,430 | 1,046,577 (1.04) | 6,179,810 | 110,105 (1.78) |
| 2009 | 105,642,368 | 1,210,654 (1.15) | 5,727,951 | 63,948 (1.12) |
| 2010 | 118,735,769 | 1,397,009 (1.18) | 5,556,431 | 365,098 (6.57) |
| 2011 | 125,611,367 | 1,479,644(1.18) | 5,067,064 | 306,168 (6.04) |
| 2012 | 141,210,555 | 2,637,164 (1.87) | 4,979,719 | 402,129 (8.08) |
| 2013 | 164,107,155 | 2,820,575 (1.72) | 5,010,128 | 371,663 (7.42) |
| 2014 | 153,550,321 | 2,409,392(1.57) | 4,955,828 | 390,324 (7.88) |
| 2015 | 159,328,055 | 2,626,748 (1.65) | 4,247,488 | 437,671 (10.30) |
| 2016 | 168,023,645 | 3,054,119 (1.82) | 4,597,335 | 384,003 (8.35) |
| 2017 | 179,845,246 | 3,404,957 (1.89) | 4,681,701 | 405,391 (8.66) |
| 2018 | 193,085,412 | 3,720,442 (1.93) | 4,720,880 | 418,377 (8.86) |
| 2019 | 207,605,698 | 4,105,019 (1.98) | 5,004,771 | 417,003 (8.33) |
| 2020 | 217,424,485 | 4,826,183 (2.22) | 4,945,008 | 324,324 (6.56) |
| 2021 | 286,294,583 | 7,488,913(2.62) | 7,497,876 | 116,262 (1.55) |

N/A=not available.

**Supplemental Table 4: The number of outpatient prescriptions in ITCWM hospitals, 2012-21**

| **Year** | **TCM Prescriptions** | | | **Antimicrobial prescriptions** | | |
| --- | --- | --- | --- | --- | --- | --- |
|  | **Total (%)** | **ITCWM** | | **Total (%)** | **ITCWM** | |
|  |  | **Number** | **Proportion (%)** |  | **Number** | **Proportion**  **(%)** |
| 2012 | 15.12 | 11,254,239 | 30.00 | 16.39 | 4,040,018 | 10.77 |
| 2013 | 18.66 | 13,987,373 | 34.62 | 16.76 | 5,405,836 | 13.38 |
| 2014 | 18.61 | 17,087,156 | 35.88 | 15.23 | 6,351,983 | 13.34 |
| 2015 | 19.24 | 19,476,899 | 37.75 | 14.71 | 6,794,688 | 13.17 |
| 2016 | 19.21 | 21,324,531 | 38.85 | 14.44 | 6,328,204 | 11.53 |
| 2017 | 18.62 | 23,655,368 | 38.78 | 15.64 | 6,862,335 | 11.25 |
| 2018 | 18.95 | 21,902,224 | 37.35 | 12.78 | 6,896,491 | 11.76 |
| 2019 | 17.67 | 24,053,302 | 37.59 | 12.18 | 8,130,608 | 12.71 |
| 2020 | 18.84 | 21,130,380 | 38.99 | 10.45 | 5,816,423 | 10.73 |
| 2021 | 27.21 | 29,057,131 | 43.27 | 11.26 | 6,944,842 | 10.34 |

**Supplemental Table 5: Changes in the use of hospital beds in ITCWM hospitals, 2003-21**

| **Year** | **Turnover rate of hospital beds** | | **Hospital bed occupancy rate** | | **Average length of hospital stay** | |
| --- | --- | --- | --- | --- | --- | --- |
|  | **Total (%)** | **ITCWM (%)** | **Total (%)** | **ITCWM (%)** | **Total (%)** | **ITCWM (%)** |
| 2003 | N/A | 19.53 | 65.29 | 63.35 | 10.35 | 11.31 |
| 2004 | N/A | 19.96 | 68.29 | 67.41 | 10.17 | 11.90 |
| 2005 | N/A | 20.31 | 70.35 | 67.96 | 10.17 | 11.57 |
| 2006 | N/A | 20.92 | 72.62 | 70.17 | 10.27 | 11.87 |
| 2007 | 25.10 | 24.39 | 78.24 | 76.91 | 10.78 | 10.96 |
| 2008 | 26.46 | 24.13 | 81.45 | 80.23 | 10.72 | 11.25 |
| 2009 | 28.07 | 26.25 | 84.75 | 82.06 | 10.52 | 10.87 |
| 2010 | 28.99 | 26.08 | 86.7 | 82.82 | 10.49 | 10.81 |
| 2011 | 30.18 | 26.85 | 88.48 | 83.41 | 10.30 | 10.86 |
| 2012 | 31.92 | 27.45 | 90.08 | 85.90 | 10.01 | 10.82 |
| 2013 | 31.90 | 28.00 | 88.99 | 85.72 | 9.84 | 10.94 |
| 2014 | 32.30 | 27.80 | 88.05 | 84.21 | 9.65 | 10.59 |
| 2015 | 31.44 | 27.28 | 85.36 | 81.52 | 9.59 | 10.44 |
| 2016 | 32.12 | 27.29 | 85.29 | 80.43 | 9.41 | 10.48 |
| 2017 | 32.30 | 27.84 | 84.97 | 80.61 | 9.30 | 10.25 |
| 2018 | 32.20 | 28.04 | 84.22 | 79.96 | 9.30 | 10.36 |
| 2019 | 32.33 | 28.20 | 83.55 | 78.08 | 9.13 | 9.87 |
| 2020 | 27.10 | 23.72 | 72.30 | 67.84 | 9.50 | 10.17 |
| 2021 | 28.40 | 25.50 | 74.55 | 71.02 | 9.20 | 9.90 |

N/A=not available.

**Supplemental Table 6: Number and share of practitioners in ITCWM outpatient departments, 2002-21**

| **Year** | **Health practitioners** | | **Licensed doctors** | | **Licensed assistant doctors** | |
| --- | --- | --- | --- | --- | --- | --- |
|  | **Total** | **ITCWM (%)** | **Total** | **ITCWM (%)** | **Total** | **ITCWM (%)** |
| 2002 | 6544 | 1711 (26.15) | 3327 | 802 (24.11) | 397 | 109 (27.46) |
| 2003 | 6270 | 1472 (23.48) | 3150 | 677 (21.49) | 380 | 103 (27.11) |
| 2004 | 6399 | 1746 (27.29) | 3146 | 764 (24.28) | 344 | 105 (30.52) |
| 2005 | 6140 | 1471 (23.96) | 2941 | 667 (22.68) | 311 | 73 (23.47) |
| 2006 | 6530 | 1551 (23.75) | 3102 | 684 (22.05) | 374 | 88 (23.53) |
| 2007 | 7275 | 1634 (22.46) | 3540 | 710 (20.06) | 388 | 90 (23.20) |
| 2008 | 7425 | 1563 (21.05) | 3613 | 648 (17.94) | 426 | 107 (25.12) |
| 2009 | 8309 | 1610 (19.38) | 3952 | 675 (17.08) | 486 | 120 (24.69) |
| 2010 | 9420 | 1882 (19.98) | 4554 | 807 (17.12) | 497 | 119 (23.94) |
| 2011 | 10,235 | 2070 (10.22) | 5083 | 93 (18.45) | 538 | 147 (27.32) |
| 2012 | 12,083 | 2546 (21.07) | 6020 | 1179 (19.58) | 563 | 146 (25.93) |
| 2013 | 13,530 | 2694 (19.91) | 6812 | 1208 (17.73) | 590 | 146 (24.75) |
| 2014 | 14,981 | 3001 (20.03) | 7800 | 1391 (17.83) | 604 | 144 (23.84) |
| 2015 | 17,157 | 3099 (18.06) | 9119 | 1447 (15.87) | 665 | 149 (22.41) |
| 2016 | 19,927 | 3617 (18.15) | 10,505 | 1671 (15.91) | 769 | 171 (22.24) |
| 2017 | 25,704 | 4104 (15.97) | 13,534 | 1872 (13.83) | 1022 | 178 (17.42) |
| 2018 | 31,619 | 4912 (15.53) | 16,506 | 2173 (13.16) | 1419 | 249 (17.55) |
| 2019 | 35,255 | 5508 (15.62) | 18,565 | 2411 (12.99) | 1765 | 297 (16.83) |
| 2020 | 38,403 | 6107 (15.90) | 20,448 | 2747 (13.43) | 1916 | 309 (16.13) |
| 2021 | 41,562 | 6256 (15.05) | 22,155 | 2738 (12.36) | 2162 | 325 (15.03) |

**Supplemental Table 7: Changes in projects of ITCWM discipline by supported NSFC, 2002-20**

| Year | Number of projects | Proportion  (%) | Funding amount  (thousand USD) | Proportion  (%) |
| --- | --- | --- | --- | --- |
| 2002 | 57 | 1.65 | 1563.24 | 0.81 |
| 2003 | 45 | 1.30 | 1447.60 | 0.75 |
| 2004 | 52 | 1.50 | 1160.59 | 0.60 |
| 2005 | 54 | 1.56 | 1795.91 | 0.93 |
| 2006 | 64 | 1.85 | 2091.29 | 1.09 |
| 2007 | 67 | 1.94 | 2697.36 | 1.40 |
| 2008 | 87 | 2.52 | 4163.07 | 2.16 |
| 2009 | 102 | 2.95 | 3961.04 | 2.06 |
| 2010 | 139 | 4.02 | 5213.59 | 2.71 |
| 2011 | 218 | 6.30 | 12933.65 | 6.72 |
| 2012 | 251 | 7.26 | 17132.95 | 8.91 |
| 2013 | 240 | 6.94 | 16391.73 | 8.52 |
| 2014 | 239 | 6.91 | 16241.26 | 8.44 |
| 2015 | 268 | 7.75 | 14861.93 | 7.72 |
| 2016 | 280 | 8.09 | 16005.80 | 8.32 |
| 2017 | 309 | 8.93 | 17539.78 | 9.12 |
| 2018 | 300 | 8.67 | 18269.85 | 9.50 |
| 2019 | 323 | 9.34 | 18115.19 | 9.42 |
| 2020 | 342 | 9.89 | 20092.23 | 10.44 |
