# Supplementary figures and images for: Progress and challenges in integrated traditional Chinese and western medicine in China from 2002 to 2021
Source: Front Pharmacol. 2024 Sep 6;15:1425940. doi: 10.3389/fphar.2024.1425940 (PMC11412861; doi:10.3389/fphar.2024.1425940)

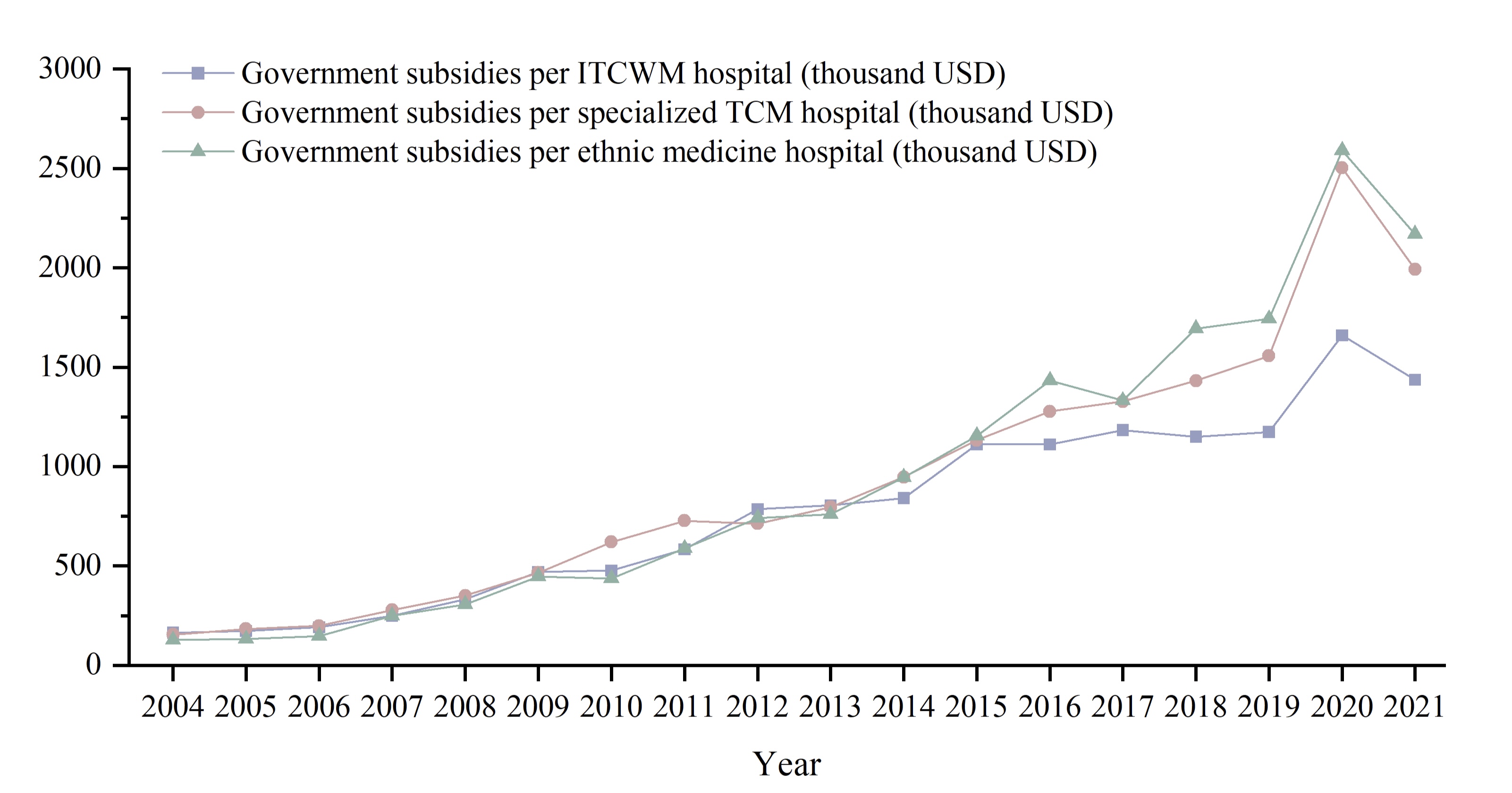

Supplement: Supplementary file 2 [file Image1.TIF]
